# Triptolide with hepatotoxicity and nephrotoxicity used in local delivery treatment of myocardial infarction by thermosensitive hydrogel

**DOI:** 10.1186/s12951-023-01980-6

**Published:** 2023-07-17

**Authors:** Kun Wang, Ke Zhu, Ziyang Zhu, Fuqiang Shao, Ruijie Qian, Chenyang Wang, Haiqing Dong, Yongyong Li, Zairong Gao, Jun Zhao

**Affiliations:** 1 Department of Nuclear Medicine, Shanghai East Hospital, School of medicine, Tongji University, Shanghai, 200120 China; 2 Department of Nuclear Medicine, Union Hospital, Tongji Medical College, Hubei Key Laboratory of Molecular Imaging, Huazhong University of Science and Technology, Wuhan, 430022 Hubei China; 3 Department of Nuclear Medicine, The First People’s Hospital of Zigong, Zigong, 643099 Sichuan China; 4 Department of Interventional Radiology, The First Affiliated Hospital of Zhengzhou University, Zhengzhou, 450052 China; 5 The Institute for Biomedical Engineering & Nano Science, School of Medicine, Tongji University, Shanghai, 200092 China

**Keywords:** Myocardial infarction, Triptolide, Pluronic F127, PLGA, Macrophage polarization, Inflammatory response

## Abstract

Myocardial infarction (MI) resulting from coronary artery occlusion is the leading global cause of cardiovascular disability and mortality. Anti-inflammatory treatment plays an important role in MI treatment. Triptolide (TPL), as a Chinese medicine monomer, has a variety of biological functions, including anti-inflammatory, anti-tumor, and immunoregulation. However, it has been proved that TPL is poorly water soluble, and has clear hepatotoxicity and nephrotoxicity, which seriously limits its clinical application. Herein, we designed a long-acting hydrogel platform (TPL@PLGA@F127) for MI treatment by intramyocardial injection. First, we found that the inflammatory response and immune regulation might be the main mechanisms of TPL against MI by network pharmacology. Subsequently, we prepared the hydrogel platform (TPL@PLGA@F127) and tested its effects and toxicity on normal organs in the early stage of MI (3 days after MI-operation). The results showed that TPL@PLGA@F127 could not only promote “repair” macrophages polarization (to M2 macrophage) by day 3 after MI, but also has a long-lasting anti-inflammatory effect in the later stage of MI (28 days after MI-operation). Additionally, we proved that TPL@PLGA@F127 could attenuate the toxicity of TPL by releasing it more slowly and stably. Finally, we observed the long-term effects of TPL@PLGA@F127 on MI and found that it could improve cardiac function, depress the myocardial fibrosis and protect the cardiomyocytes. In summary, this study indicated that TPL@PLGA@F127 could not only enhance the therapeutic effects of TPL on MI, but also attenuate the hepatotoxicity and nephrotoxicity, which established a strong foundation for the clinical application of TPL for MI.

**Supplementary Information:**

The online version contains supplementary material available at 10.1186/s12951-023-01980-6.

## Introduction

Myocardial infarction (MI) resulting from coronary artery occlusion is the leading global cause of cardiovascular disability and mortality [1]. Myocardial infarction involves a variety of pathological factors, among which the inflammatory response is an important feature [2]. Inflammation and inflammatory cell infiltration are important indicators of myocardial injury [3]. During the early stage of MI, a large number of inflammatory cells (inflammatory macrophages, and neutrophil among others) are recruited, and these cells secrete a variety of inflammatory mediators, contributing to the exacerbation of myocardial injury. In addition, excessive inflammation can lead to poor ventricular remodeling and adversely affect cardiac function recovery [2]. Therefore, the appropriate use of anti-inflammatory therapy after myocardial infarction may be the key step in its treatment.

Triptolide (TPL) is a chemical compound with pharmacological activity isolated from the traditional Chinese medicine *Tripterygium wilfordii*. Its chemical structure is shown in Fig. S1. Studies have shown that TPL has a variety of biological functions, including anti-inflammatory, anti-tumor, and immunoregulation among others [4]. Wen et al. [5] found that TPL significantly attenuated cardiac inflammation and fibrosis through suppressing the activity and the expression of NF-κB, resulting in improved left ventricle function in experimental diabetic cardiomyopathy. It is unclear whether TPL can be used to treat MI, but we propose that it may have an excellent therapeutic effect on MI based on the therapeutic application of TPL on cardiovascular diseases [5–7]. However, systemic TPL administration may cause obvious hepatotoxicity and nephrotoxicity, which has seriously limited its clinical application [8, 9], Moreover, TPL has poor water solubility, resulting in low bioavailability [10]. Therefore, developing new TPL preparations to achieve the therapeutic effects of synergism and attenuation of toxicity is a challenge that must be solved for the clinical application of TPL.

One option to address the hepatotoxicity and nephrotoxicity of TPL is local sustained release in the heart using in situ injection [11]. Polylactic-co-glycolic acid (PLGA) is a biodegradable copolymer approved by the U.S. Food and Drug Administration (FDA). The PLGA nanoparticle can encapsulate a variety of drugs to increase bioavailability and provide sustained drug release [12]. However, one of the shortcomings of PLGA nanoparticles is the occurrence of drug burst release, and excessive drug release in the burst phase may be toxic [13]. In addition, Direct myocardial injection of PLGA nanoparticles also involves the problem of whether these nanoparticles can reside in the myocardium. It has been reported that up to 90% of stem cells injected directly into the myocardium can be lost within a day [14]. To solve the problem of implanted cells loss in the myocardium, a combined hydrogel implantation method has been normally applied. We hypothesized that the nanoparticles loss from the myocardium could also be solved by this method. Hydrogel is the most commonly used material in heart tissue engineering and its intramyocardial injection can provide mechanical support to improve the compliance of the ventricular wall [15]. In addition, hydrogel is also a good platform for drug sustained drug release for myocardial tissue repair, thus we speculated that the use of dual sustained release of hydrogel and PLGA nanoparticles could solve the problem that the slow-release curve of PLGA nanoparticles was not sufficiently smooth. Pluronic F127 is a form of tricopolymer poly (ethylene oxide)-poly (propylene oxide)-poly (ethylene oxide) (PEO-PPO-PEO), and has been approved by the FDA in clinical practice [16]. On the basis of its special structure, F127 can transform to the gel state at body temperature and maintain the liquid state at a lower temperature. The drug loaded in F127 hydrogel is normally released within 4 days, which can prevent the drug burst release of PLGA nanoparticles at the initial stage of drug release [17]. Therefore, F127 gel and PLGA nanoparticles may be an excellent choice for in situ TPL myocardial delivery to attenuate the toxicity.

**Scheme 1:**
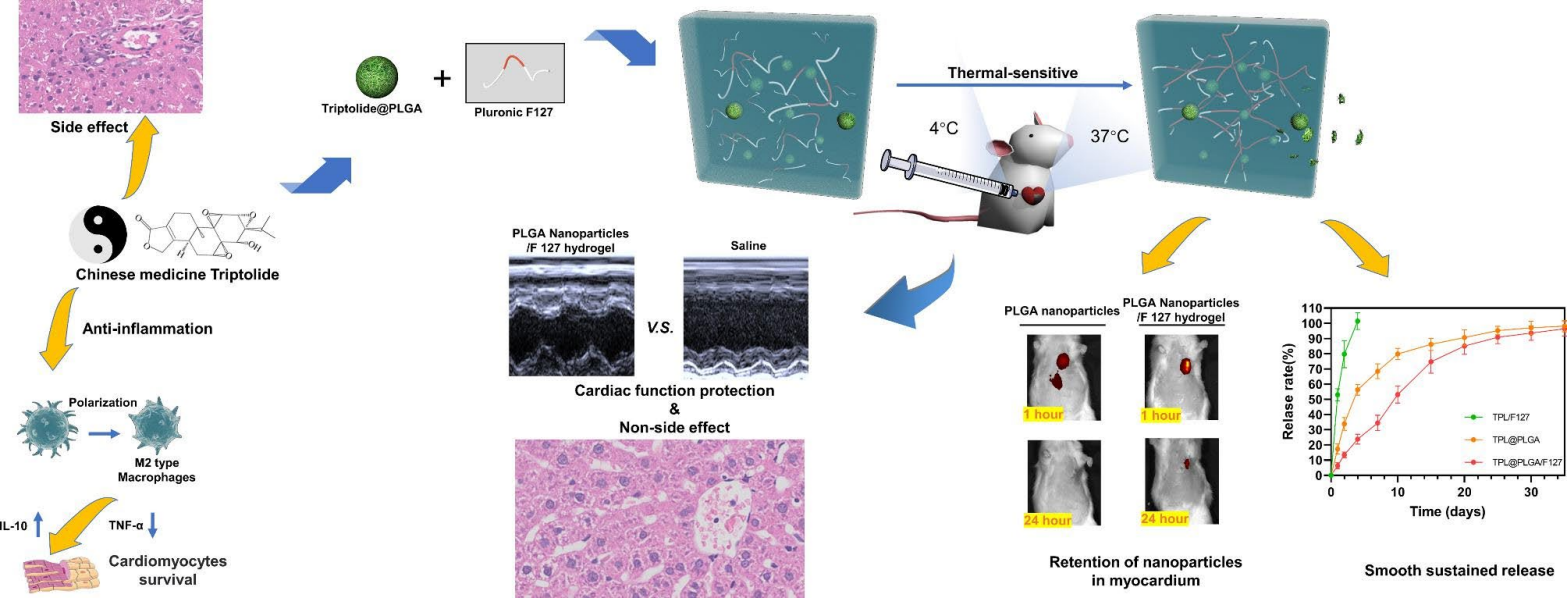
Thermal-sensitive hydrogel platform (TPL@PLGA@F127) enables TPL release more slowly and stably, which not only enhances the therapeutic effects on myocardial infarction (MI), but also attenuates the hepatotoxicity and nephrotoxicity

In this study, we first assessed the possibility and mechanism of TPL against MI by network pharmacology and subsequently designed a dual sustained release system (TPL@PLGA@F127) which consisted of TPL@PLGA and Pluronic F127. TPL@PLGA nanoparticles would be well-dispersed in the hydrogel and could achieve long-term TPL release. Additionally, the hydrogel drug delivery platform (TPL@PLGA@F127) could avoid the sudden drug release caused by PLGA and increase the retention time of TPL in the infarcted myocardium, which would provide a sustainable support for TPL treatment. Finally, we assessed the early-phase (3 days after MI-operation) and later-stage (28 days after MI-operation) effects on MI and the toxicity to normal organs in the early stage by in vivo and in vitro experiments. Overall, through in situ myocardial injection, we believe that TPL@PLGA@F127 can achieve not only long-term and stable TPL release, but also MI treatment and attenuation of hepatotoxicity and nephrotoxicity (Scheme 1).

## Materials and methods

### Network pharmacologic analysis of TPL against MI

Firstly, the genes related to MI were acquired from GeneCards (http://www.genecards.org), Drugbank (https://go.drugbank.com/), OMIM (https://www.omim.org/), CTD (http://ctdbase.org/voc.go?type=chem) and the genes of TPL were got from TCMSP (http://lsp.nwu.edu.cn/tcmsp) and GeneCards online database. With Venny 2.1 (http://bioinfogp.cnb.csic.es/tools/venny/), we got the Venn diagram and identified the interaction genes between the MI and TPL-related genes. Subsequently, the interaction targets were further analyzed. Protein-protein interaction (PPI) network was built by the STRING online tool (https://string-db.org) and the confidence score was over 0.9. Then, the overlapping genes were imported into DAVID online platform (https://david.ncifcrf.gov) to carry out the GO enrichment analysis and the top 20 biological processes (BP), cellular components (CC), and molecular functions (MF) were selected based on the degree value. Finally, the compound-target-biological processes (C-T-B) network was built by the Cytoscape v3.7.2 software.

### The preparation and characteristics of TPL@PLGA

The TPL@PLGA nanoparticle was prepared by a double emulsion (water/oil/water) method [18]. Firstly, we mixed TPL (40 mg/ml, Solarbio, China) with 20 mg PLGA [Poly (D,l-lactide-co-glycolide, with a co-monomer ratio of 75/25 (lactic/glycolic acid), Mw 4000-15,000,Mecklin, shanghai] in 2ml dichloromethane (DCM) and then sonicated the solution under ice-water condition for 10 min. Subsequently, we added the emulsion into 0.5% w/v polyvinyl alcohol (PVA) dropwise and sonicated under ice-water condition for 20 min. Then, the solution was added into 25ml dimethyl carbinol (2%) and stringed for 4 h at room temperature. TPL@PLGA were purified by centrifugation (12,000 rpm, 15 min) to remove free TPL, and micronized PLGA. DIR@PLGA was prepared by the similar method. The dynamic diameters and zeta potential were measured by the Zeta PALS (Malvern Instruments, UK). Transmission electron microscopy (TEM) was performed after staining the samples negatively with 1% phosphotungstic acid.

### The preparation and characteristic of TPL@PLGA@F127

The method of preparation was described previously [19]. Briefly, the TPL@PLGA was resuspended in distilled water and then mixed with the F127 solution until the concentration of F127 is 25% w/v and then stirred in 4℃ for 10 min to ensure that NPs were evenly distributed in the F127 solution. TPL@F127 was prepared by the similar method. Scanning Electron Microscope (SEM) images of F127 and TPL@PLGA@F127 were performed on SEM (QUANTA200).

The temperature-induced phase transition behaviors of F127 or TPL@PLGA@F127 were measured based on the methods described previously [20]. Briefly, the storage modulus (G′) and loss modulus (G″) were measured on the temperature range (15 to 40 °C) at an angular frequency of 1 rad/s. In vitro release experiments were carried out as previously described. The TPL@PLGA and TPL@PLGA@F127 were putted into a trans-well insert and bathed in PBS to quantify the release of TPL, and the pore size of trans-well insert is 8 μm. The PBS was changed and collected to detect the concentration of TPL by HPLC. Each assay was performed in 2 ml of PBS in a 24-well plate at 37 °C.

In vitro release experiments were performed as described in other studies [19, 21]. The content of TPL was measured by high-performance liquid chromatography (HPLC) equipped with an ultraviolet detector. The encapsulation efficiency (EE) was calculated using the following formula: EE% = (W_encapsulated_/W_total_) × 100%, where W_encapsulated_ and W_total_ represented the content of drug after and before elution.

### In vitro cell experiment

The effect of TPL@PLGA@F127 on the macrophages was identified. The macrophage cell line (RAW 264.7) was cultured in DMEM medium with 10% fetal bovine serum (FBS, VivaCell, Shanghai, China) and 1% penicillin/streptomycin (Shanghai Chuanqiu Biotechnology Co.,Ltd, China.) in 37℃ and 5% CO_2_. CCK8 kit (New Cell & Molecular Biotech, China) was used to monitor the cell viability of RAW 264.7. Briefly, the cell was incubated with TPL, TPL@PLGA, TPL@PLGA@F127 with different concentration of TPL for 24 h in 96-well plates. Then the drug was removed and the cell was incubated with serum-free medium with 10% CCK8 for 2 h. Finally, 450 nm was used to measure the absorbance of viable cells.

The effect of F127 and TPL@PLGA@F127 on the cardiocyte was identified by Calcein-AM/PI Live-Dead Cell Staining Kit (Shanghai zcibio technology Co.,Ltd, China). Firstly, H9C2 cell line (Procell Life Science&Technology Co.,Ltd, China) was were incubated with F127 and TPL@PLGA@F127 for 24 h, and then incubated with Calcein-AM/PI Live-Dead Cell Staining Kit for 30 min and observed by fluorescence microscopy. Finally, the cells were frozen by Cell Freezing Medium (Shanghai Chuanqiu Biotechnology Co.,Ltd, China.).

To investigate the effect of TPL, PLGA, F127 and TPL@PLGA@F127 on macrophage phenotype, immunofluorescence Immunofluorescence (IF) was carried out to show the expression of CD206 and CD86. TPL, PLGA, F127 were incubated with RAW 264.7 for 24 h and then incubated with anti-CD86 Polyclonal Antibody (1:1000, Solarbio, Beijing, China) and anti-CD206 antibody (1:2000, Proteintech, Wuhan, China). And then the cells were incubated with FITC Conjugated AffiniPure Goat Anti-rabbit IgG (H + L) (1:100, boster, Wuhan, China) and CY3 Conjugated AffiniPure Goat Anti-mouse IgG (H + L) (1:100, boster, Wuhan, China). The expression was observed using the 570 nm and 520 nm lasers for red and green fluorescence, and we obtained images at oil immersion magnifications of ×400. Then the effect of TPL@PLGA and TPL@PLGA@F127 in different time point on macrophage phenotype were tested with the similar methods.

Besides, the effect of TPL on the polarization of macrophages was verified by flow cytometry. Firstly, the macrophage cells (Raw 264.7) were incubated with TPL for 24 h, then the cells were obtained by centrifugation and anti-CD16/CD32 (Elabscience Biotechnology Co.,Ltd, China) was used to block Fcγ-receptors. Then the cells were stained with PE Anti-Mouse CD206 Antibody (Biorbyt, Cambridge, UK) and got the result by BD FACS Canto flow cytometer.

### MI model preparation and treatment of with TPL@PLGA@F127

Huazhong University of Science and Technology’s Institutional Animal Care and Use Committee supervised and approved all animal studies at Tongji Medical College. Weitong Lihua Laboratory Animal Center (Beijing, China) provided 200 g ± 10 g male rats for this study. The preparation of MI model was prepared as described previously [22]. Briefly, after the rat was anesthetized with 6 mg/kg sodium pentobarbital, the left anterior descending coronary artery (LAD) was ligated.

MI Rats were injected intramyocardially with 100 µl of gel drug (the concentration of TPL was 5 mg/ml) in 30 min after the operation. the rats were followed up 3 days and 4 weeks after MI-operation and were evaluated the left ventricle (LV) function by structural remodeling assessment and echocardiography.

### Echocardiographic Assessment

left ventricular function of rats in 28 days after MI-operation was assessed by Philips EPIQ5 system.

Firstly, rats were anesthetized with isoflurane. And then left ventricular internal diastolic diameter, short systolic fraction, left ventricular ejection fraction, left ventricular end-diastolic volume, left ventricular internal systolic dimensions and left ventricular end-systolic volume were measured in parasternal long-axis views.

### Histological experiments and analysis

In the day 3 after the injection of saline, TPL@PLGA, TPL@F127, TPL@PLGA@F127, the main organs (heart, liver, spleen, lung and kidney) were taken out for HE staining to identify the effect of every group [TPL(I.P.), TPL@PLGA@F127, TPL@PLGA, TPL@F127, saline, sham] on the main organs of MI rats.

In the day 28 after the treatment of rats MI model, the myocardial tissue from different groups (TPL@PLGA@F127, TPL@PLGA, TPL@F127, saline, sham) was stained by immunofluorescence, Masson, Sirius red and tunnel to assess the inflammatory response, myocardial fibrosis and construction and apoptosis of myocardial cells.

### Western blot

In day 28 after the treatment of rats MI model, the myocardial tissue from different groups (TPL@PLGA@F127, TPL@PLGA, TPL@F127, saline) was used to extracted protein by ExKine™ Total Protein Extraction Kit (Abbkine Scientific, Wuhan, China). A 10% SDS-PAGE (EpiZyme, China) was used for electrophoresis and separated the protein. Subsequently, the protein was transferred onto PVDF membranes in rapid transfer buffer (20X, New Cell & Molecular Biotech, China) and was blocked in protein-free rapid blocking buffer (EpiZyme, Shanghai, China) for 1 h. then, the PVDF membrane was incubated with primary antibodies for 12 h at 4 °C and HRP-coupled secondary antibody solution (rabbit anti-mouse or goat anti-rabbit IgG, 1:10 000, Boster, Wuhan, China) for 2 h. The primary antibodies are as follows: Collagen I (1:1500, Proteintech, Wuhan, China), Collagen III (1:1000, Proteintech, Wuhan, China), Cleaved Caspase 3(1:500, Abclonal, Wuhan, China), TNF-α (1:2000, Bioss, USA), IL-10 (1:2000, Proteintech, Wuhan, China), GAPDH (1:2000, Abclonal, Wuhan, China), β-tubulin (1:2000, Proteintech, Wuhan, China). Finally, ECL kit (Biosharp, Anhui, China) for 3 min, and imaged on a Visionwork system.

### Statistical analysis

All the values were presented as mean and standard deviation. The results were analyzed by one-way analysis of variance analysis (ANOVA) in GraphPad Prism 9.1 (GraphPad). Probability values P < 0.05 were considered significant.

### Results

#### Network pharmacologic analysis of TPL against MI

The therapeutic mechanism of natural drugs on diseases is extraordinarily complex, with various pathways and targets together being the main feature. Network pharmacology is a form of bioinformatics method, by which we can predict effective drug targets, understand the relationship between drugs and diseases, and explore the mechanism of drugs [23]. Therefore, we aimed to assess the possibility of TPL against MI and determined the related mechanisms by network pharmacology to guide the design of the follow-up TPL-related experiments.

**Fig. 1:**
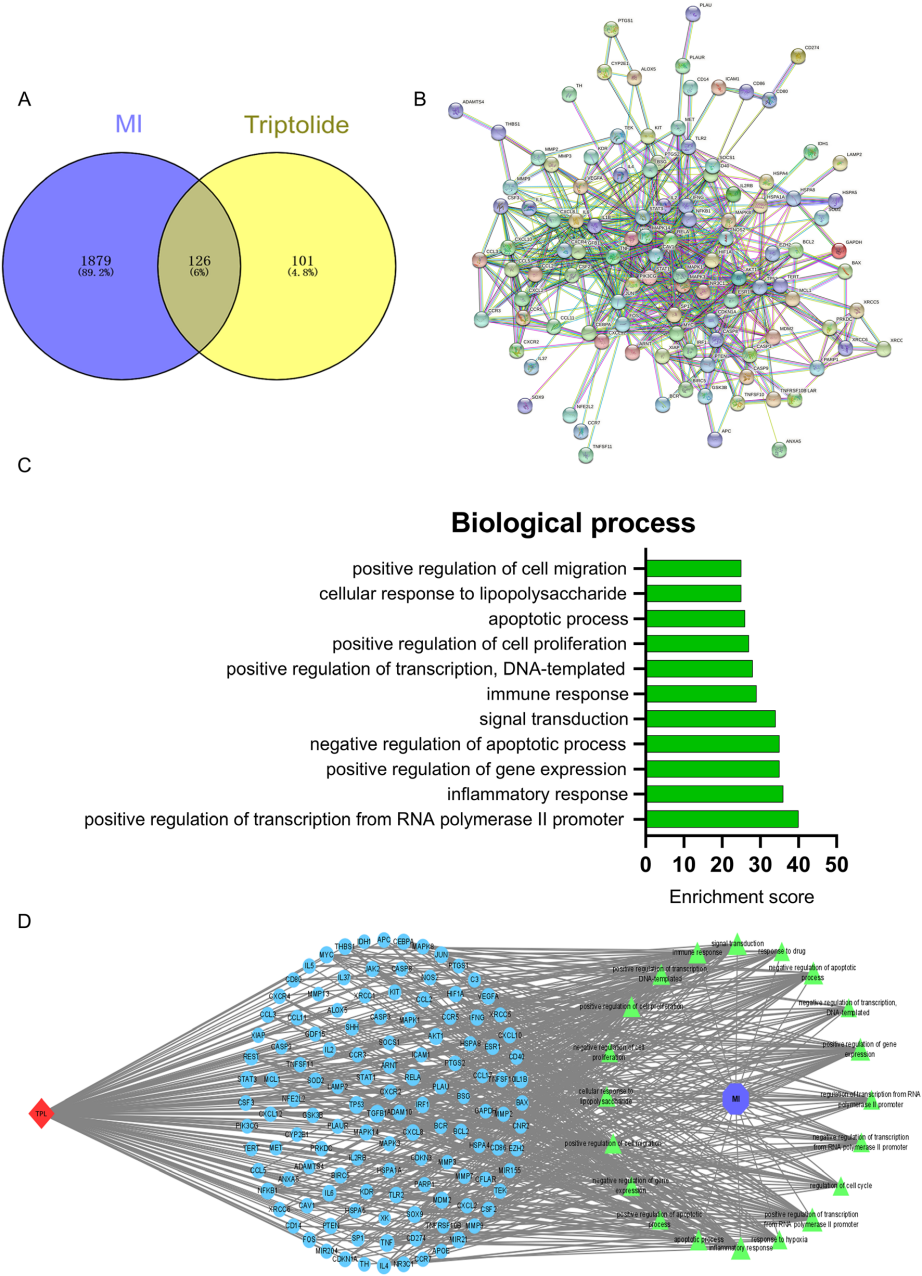
Network pharmacologic analysis of TPL on MI. (**A**) The Venn diagram of MI and TPL. (**B**) PPI network. (**C**) The biological process (BP) of TPL against MI. (**D**) the compound-target-biological processes (C-T-B) network

Firstly, we obtained 2005 genes related to MI from the online databases (GeneCards, Drugbank, OMIM, CTD; Table S1) and 227 TPL-related genes from the drug databases (TCMSP, GeneCards, table S2). Then, we obtained 126 core genes in MI treatment by defining the interaction between them (Fig. 1A, table S3). Consequently, by visualizing and analyzing the 126 core genes, we generated the PPI network (Fig. 1B), and found that many MI-related targets, including tumor necrosis factor (TNF-α), interleukin-6 (IL6), and IL-1β were located at the core of the network.

To define the detailed mechanism of TPL against MI, we performed the GO enrichment based on the 126 core targets (Fig. S2). GO enrichment includes the biological process (BP), cell component (CC), and molecular function (MF). We mainly focused on the analysis of BP (Fig. 1C), and obtained the top ten TPL-related biological processes against MI. Additionally, we found that the enrichment score of the inflammatory response, negative regulation of apoptotic process, immune response, apoptotic process was relatively higher in all biological processes (Table S4), which meant that these processes might play a core role in the process of TPL against MI. Moreover, these biological processes were also considered as the key points for MI treatment. Therefore, we hypothesized that TPL could exert a therapeutic effect on MI mainly through immune and inflammatory processes. Finally, we visualized the therapeutic processes and performed the compound-target-biological processes (C-T-B) network using Cytoscape software based on the PPI network and GO enrichment (Fig. 1D). These results suggested that TPL was possibly applied to treat MI. However, the application of TPL for the treatment of MI still required the avoidance of the significant hepatotoxicity and nephrotoxicity of TPL.

### Characteristics of TPL@PLGA@F127

**Fig. 2:**
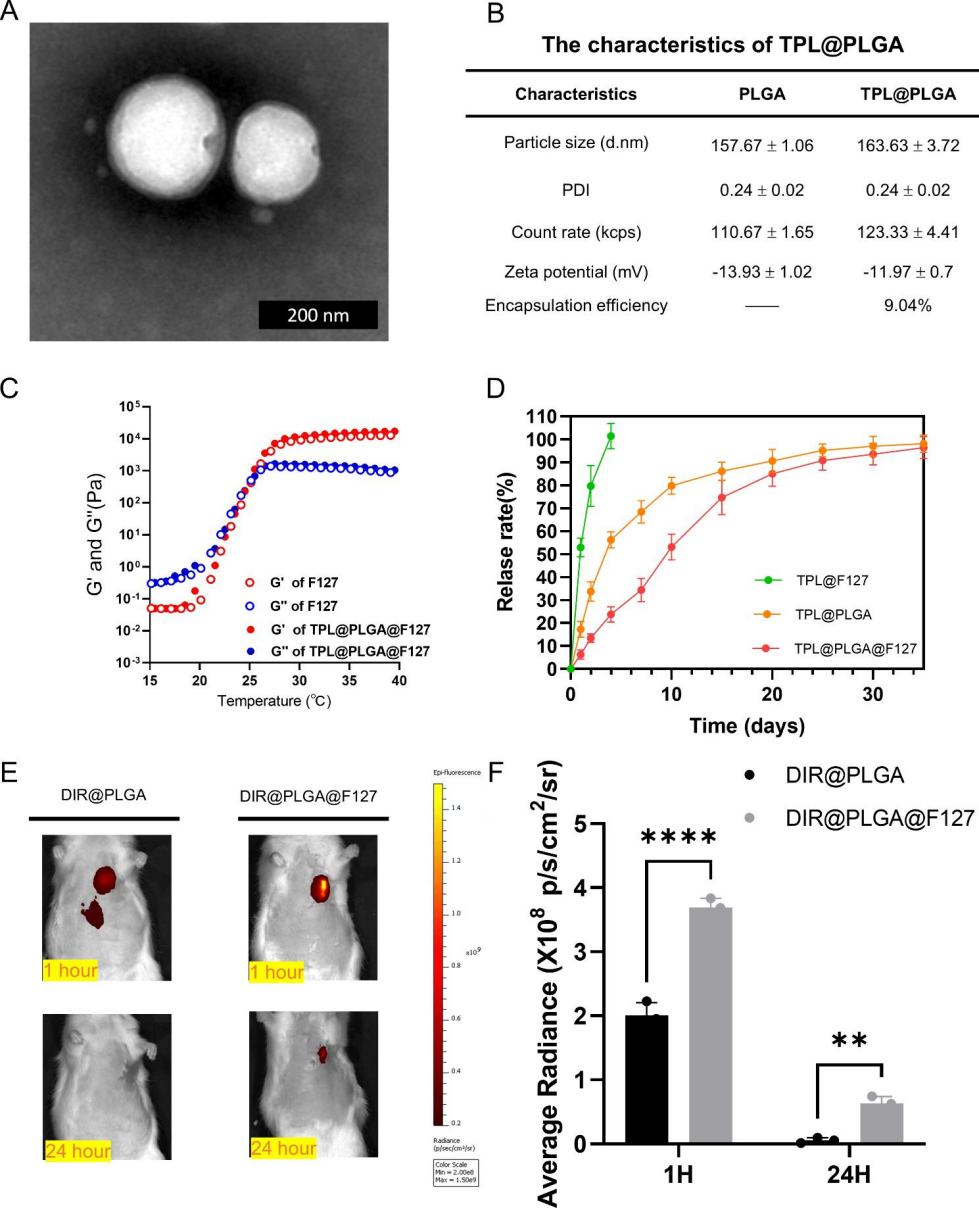
The characteristics of TPL@PLGA and TPL@PLGA@F127. (**A**) the transmission electron microscope (TEM) of TPL@PLGA. (**B**) the size, PDI, Zeta potential and encapsulation efficiency of TPL@PLGA. (**C**) Temperature dependence of the storage modulus (G′) and loss modulus (G″) of F127 and TPL@PLGA@F127. (**D**) The in vitro release of TPL@F127, TPL@PLGA, TPL@PLGA@F127. (E&F) Local retention of DIR@PLGA and DIR@PLGA@F127 and the semi-quantitative analysis of fluorescence intensity. All bars represent as means ± SD (n = 3). *P < 0.05 and **P < 0.01, ***P < 0.001

To investigate the possibility of applying TPL for MI treatment, we developed a hydrogel system for in situ TPL myocardial delivery, which consisted of PLGA nanoparticles loaded with TPL and F127 thermo-sensitive hydrogel. First, TPL@PLGA was synthesized by the double emulsion-solvent evaporation technique, which was confirmed by transmission electron microscope (TEM, Fig. 2A), and the result indicated that TPL@PLGA was of a well-defined spherical shape. Additionally, we determined the size and Zeta potential of PLGA and TPL@PLGA, and the encapsulation efficiency of TPL@PLGA. As shown in Fig. 2B, the mean hydrodynamic diameter, polydispersion index (PDI) and Zeta potential of PLGA and TPL@PLGA was 157.67 ± 1.06 vs. 163.63 ± 3.72 nm, 0.24 ± 0.02 vs. 0.24 ± 0.02, and -13.93 ± 1.02 vs. -11.97 ± 0.7mV, respectively, and encapsulation efficiency of TPL@PLGA was 9.04%. TPL@PLGA@F127 was generated by mixing TPL@PLGA and F127 gel at 4 °C. The results of Scanning Electron Microscope (SEM) of F127 and TPL@PLGA@F127 were shown in Fig. S3, which shown that they had the similar structure. The effects of temperature on the mechanical properties of TPL@PLGA@F127 were assessed (Fig. 2C), and it was found that the addition of PLGA nanoparticles in F127 did not significantly change the modulus of F127 hydrogel, and the critical temperature of the phase transition for both F127 and TPL@PLGA@F127 was 23 °C.

### ***In vitro*** release and ***in vivo*** retention of TPL in TPL@PLGA@F127 platform

As shown in Fig. 2D, compared to TPL@PLGA and TPL@PLGA@F127, TPL in TPL@F127 was released the most rapidly, and the release rate could reach nearly 100% in day 4, whereas the release rate in TPL@PLGA and TPL@PLGA@F127 were 56.21±3.53% and 23.81±3.30%, respectively. The release of TPL in TPL@PLGA@F127 was slower and more stable than in TPL@PLGA during the whole period, which because the dual release of TPL in TPL@PLGA@F127 that TPL was released from PLGA into hydrogel firstly and then released from hydrogel into infarcted myocardium. In addition, we found that there is no the burst release of TPL in TPL@PLGA@F127 compared to TPL@PLGA, indicating that F127 gel could avoid the burst release of TPL@PLGA in the initial stage, but did not change its accumulated dose of release. Subsequently, we assessed whether the F127 gel could promote the intramyocardial retention of PLGA nanoparticles during in situ injection through DIR labeling of PLGA nanoparticles, whereby DIR@PLGA and DIR@PLGA@F127 were injected into the infarcted myocardium in rats and detected near-infrared fluorescence images in in vivo at 1 and 24 h after the MI operation (Fig. 2E and F). We found that a part of the DIR@PLGA had run off from the myocardium by 1 h after the injection, whereas the DIR@PLGA@F127 was all concentrated in the injection-site. At 24 h, we found that the fluorescence intensity of DIR@PLGA was the same as the background intensity, whereas the fluorescence of DIR@PLGA@F127 could be still observed, which indicated that the F127 gel contributed to the longer in vivo PLGA nanoparticle retention.

### TPL@PLGA@F127 regulated the immune process in ***in vitro*** and ***in vivo*** experiments

**Fig. 3:**
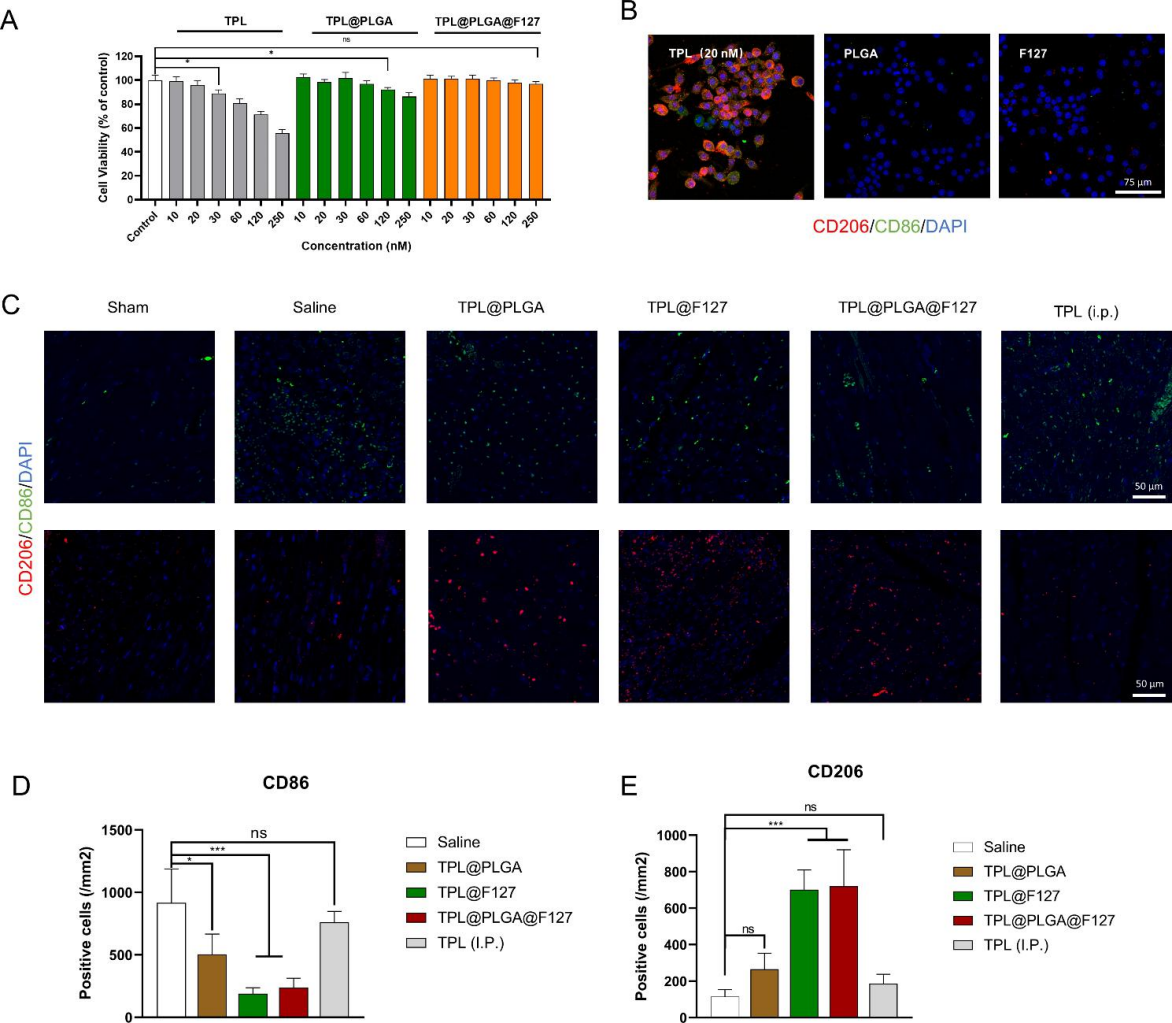
The effect of TPL@PLGA@F127 on macrophages in in vitro and in vivo experiment. (**A**) The effect on cell viability of macrophages. (**B**) The effect on the polarization of macrophages. (**C**) the immunofluorescence staining of myocardial sites in the day 3 after treatment.CD206 and CD86 were the biomarker of M2 and M1 macrophage respectively. (**D**&**E**) The semi-quantitative analysis of immunofluorescence staining. All bars represent as means ± SD (n = 3). *P < 0.05 and **P < 0.01, ***P < 0.001

From the result of network pharmacology, we hypothesized that the immune and inflammatory processes might be the key points of TPL against MI, thus we investigated the effects of TPL@PLGA@F127 on macrophages. As we can see in Fig. 3A, when the TPL concentration was over 20 nM, it could significantly inhibit macrophage viability, indicating that TPL had significant toxicity. By contrast, cells treated with TPL@PLGA or TPL@PLGA@F127 even with a higher TPL concentration, did not display significant inhibitory effects. The reason may be the low drug concentration in the cell medium due to the slow TPL release resulting from the slow PLGA and F127 gel degradation, which indicated that TPL@PLGA@F127 design might be possibly applied to attenuate the toxicity in practice.

Subsequently, we evaluated the effect of TPL on macrophage polarization and found that a TPL concentration of 20 nM down-regulated the expression of CD86 (M1 macrophage subtype marker) and up-regulated the expression of CD206 (M2 macrophage subtype marker) on the surface of macrophages (Fig. 3B), which indicated that TPL mediated macrophages polarization towards M2. Meanwhile, we also performed flow cytometry of Raw 264.7 (Fig. S4) and also found that TPL (20 nM) could increase the expression of CD206 and make macrophages polarization towards M2. Of note, TPL (20 nM) did not affect macrophage viability, therefore, we hypothesized that TPL might regulate the immune process mainly by affecting the phenotype of macrophages rather than directly inhibiting the viability.

On the basis of the above analysis, we also verified the effect of TPL@PLGA@F127 on immune regulation by in vivo experiments and performed immunofluorescence staining of infarcted myocardium at day 3 after treatment (Fig. 3C and E). The result showed that the TPL@PLGA@F127 and TPL@F127 groups had the best effect on the promotion of CD206 positive cells (M2 macrophages) and the reduction of CD86 positive cells (M1 macrophages) in infarcted myocardium compared with the other groups, and that TPL@PLGA@F127 had a similar effect as TPL@F127. The above results demonstrated that TPL@PLGA@F127 and TPL@F127 both might have an excellent effect on immune regulation by day 3 after MI operation.

Overall, TPL@PLGA@F127 had an effect of immune regulation by day 3 after the MI operation, which also conformed the result of network pharmacology.

### Biosafety assessment of TPL@PLGA@F127 in the early stage

Firstly, we evaluated the toxicity of F127 and TPL@PLGA@F127 on H9C2 cells by Calcein-AM/PI Live-Dead Cell Staining Kit and found that F127 and TPL@PLGA@F127 could not causes significant death to H9C2 (Fig. S5), indicating that the material had excellent biosafety. Then we evaluated the toxicity of TPL (intraperitoneal, i.p.) in the normal mode of administration and we were surprised found that survival rate of rats with MI is heavily influenced by TPL (i.p.). As shown in Fig. S6, half of the rats had died in the day 1 after the treatment in TPL (i.p.) group, whereas the rats in saline group were survived, which demonstrated that TPL (i.p.) would have the severe acute toxicity to the rats with MI and intraperitoneal injection might not be a rational and practical administration.

**Fig. 4:**
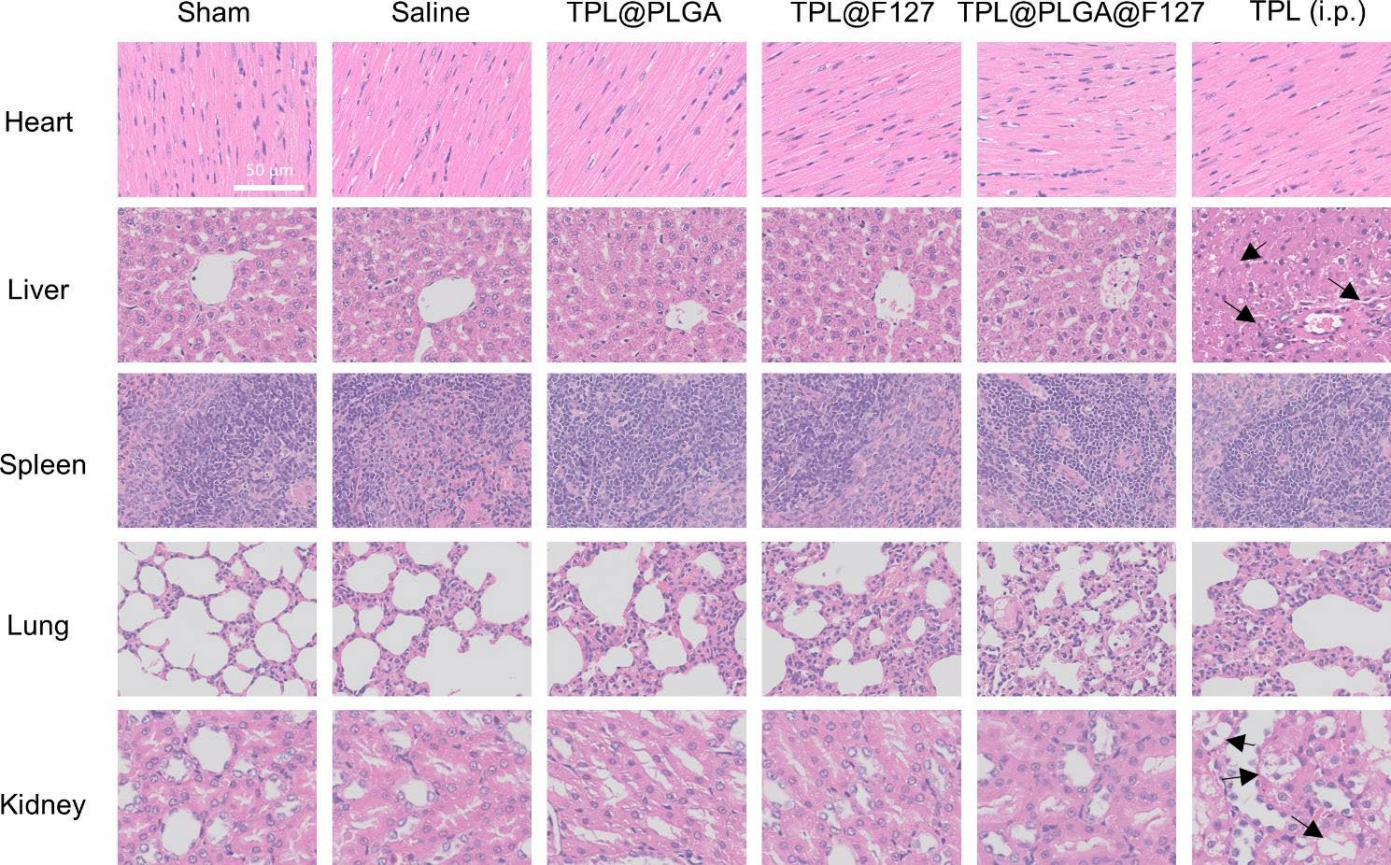
The HE staining of main organs (Heart, liver, spleen, lung, kidney, and arrows indicate abnormal)

Subsequently, we evaluated the toxicity of different treatments to the main organs. As shown in Fig. 4, we assessed the structure of the main organs in the different groups. The results showed that in the TPL (i.p.) group, there were inflammatory cell infiltration, necroptosis and disruption of cellular architecture in liver tissue (indicated by arrows). In addition, we found severe cell edema and vacuolar degeneration in kidney tissue in the TPL (i.p.) group (indicated by arrows), whereas these abnormalities were not observed in the TPL@PLGA@F127 group, indicating that the design of TPL@PLGA@F127 could attenuate the nephrotoxicity and hepatotoxicity of TPL when TPL was provided by conventional administration.

### TPL@PLGA@F127 exhibited excellent long-term anti-inflammatory effects

**Fig. 5:**
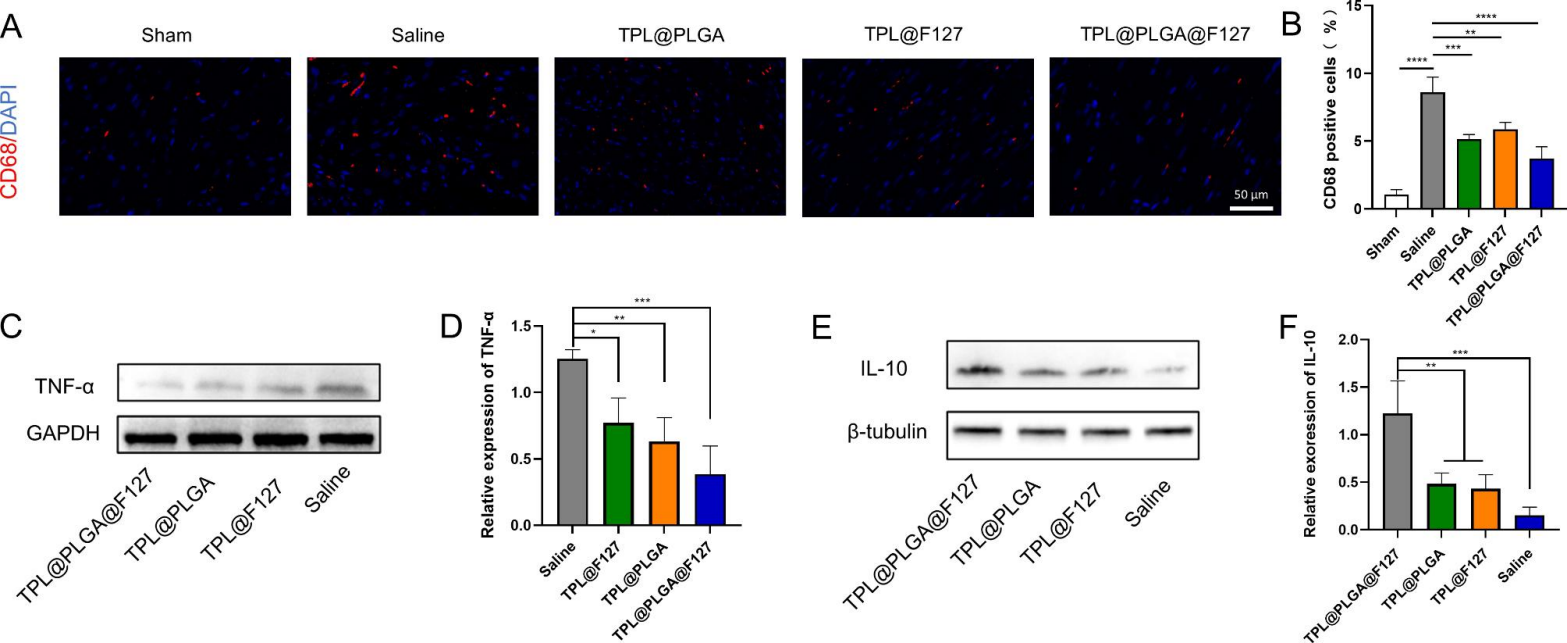
The evaluation of anti-inflammation effects on MI of TPL@PLGA@F127 in the day 28 after treatment. (A)the immunofluorescence staining of myocardial infarction sites. CD68 was the red fluorescence and the nucleus was the blue fluorescence. (B) the semi-quantitative analysis of immunofluorescence staining. (C-F) the western blot of TNF-α and IL-10 in myocardial infarction sites and the semi-quantitative analysis. All bars represent as means ± SD (n = 3). *P < 0.05 and **P < 0.01, ***P < 0.001

Network pharmacology analysis showed that TPL also influenced myocardial infarction treatment through anti-inflammation, in addition, long-term chronic prolonged inflammation after MI was an important factor in MI prognosis. Macrophages are the main inflammatory cells involved in the chronic inflammatory processes [24], therefore, we evaluated the number of macrophages in myocardium by immunofluorescence staining at the day 28 after treatment. As shown in Fig. 5A and B, CD68 was the key biomarker of macrophages. There were less macrophages infiltration in the TPL@PLGA@F127 group than in the TPL@F127 groups, which indicated that TPL@PLGA@F127 with a more sustainable release than TPL@F127 could play an anti-inflammatory role even in the later stage of MI. Additionally, there was more retention of TPL in TPL@PLGA@F127 group than TPL@PLGA group, which resulted in the more concentration of TPL in infarcted myocardium, causing that the TPL@PLGA@F127 group had the less macrophages infiltration in the later stage of MI. Besides, the result of western blot showed that there was less TNF-α and more IL-10 in the infarcted myocardium of the TPL@PLGA@F127 group (Fig. 5C-F), which also proved that the inflammation in infarcted myocardium remained depressed by TPL@PLGA@F127 even in the later stage of MI.

### TPL@PLGA@F127 suppressed cardiomyocytes apoptosis and protected the myocardial microstructure

**Fig. 6:**
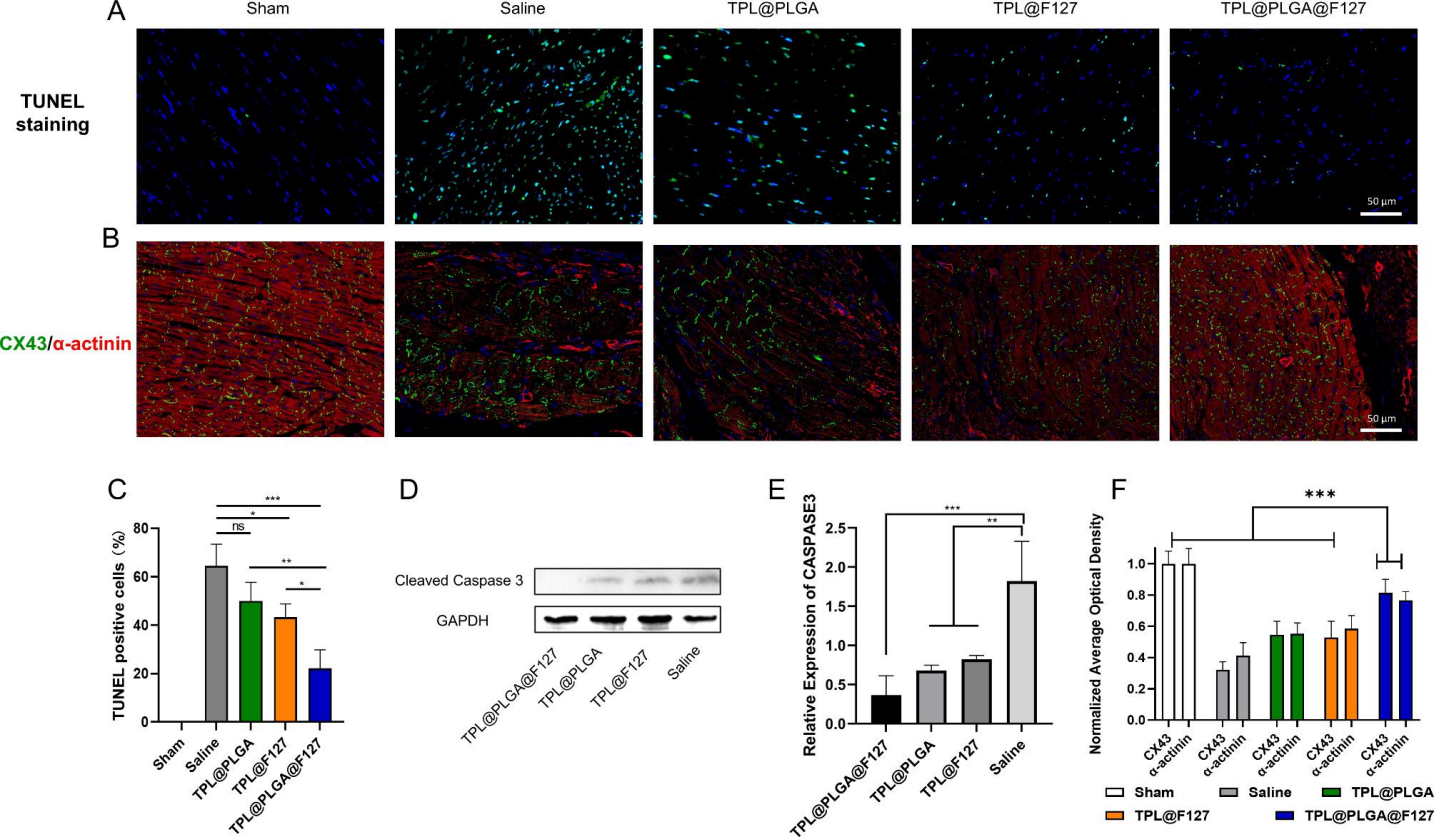
Assessment of myocardial structure and cardiomyocyte survival by pathological staining. (**A**&**C**) the TUNEL staining of the ventricular anterior wall and semi-quantitative analysis. (**B**&**F**) Representative immunofluorescence images co-stained by CX43 (green) and α-actinin (red) and their semi-quantitative analysis. (**D**&**E**) the western blot results of cleaved Caspase 3 and the semi-quantitative analysis. All bars represent as means ± SD (n = 3). *P < 0.05 and **P < 0.01, ***P < 0.001

In addition, we evaluated cardiomyocyte apoptosis by TUNEL staining and western blot of infarcted myocardium and found that TPL@PLGA@F127 group had the lowest TUNEL-positive rate compared with the other groups, which verified that TPL@PLGA@F127 could promote cardiomyocyte survival after MI (Fig. 6A-C). The results of western blot also showed that TPL@PLGA@F127, TPL@PLGA, and TPL@F127 all reduced the expression of cleaved caspase 3, with TPL@PLGA@F127 having the greatest effect (Fig. 6D and E). In addition, we detected myocardial microstructure changes by immunofluorescence staining, and α-actinin and connexin 43 (CX43) were detected as the main biomarker of myocardial structure. α-actinin is a key protein of the cardiomyocyte cytoskeleton, and CX43 can transmit electrical signals between cardiomyocytes [25]. The result in the saline group showed that the expression of CX43 and α-actinin was significantly decreased, and CX43 was located around the nucleus and could thus not play the role of electrical conduction (Fig. 6B). There was no CX43 in some infarcted myocardium. By contrast, there was higher CX43 expression and more functional localization in the TPL@PLGA@F127, TPL@PLGA and TPL@F127 group compared with the saline group in infarcted myocardium (Fig. 6B). We assessed the average optical density of CX43 and α-actinin in immunofluorescence staining, and found there were the highest expression in TPL@PLGA@F127 compared with TPL@PLGA and TPL@F127 group (Fig. 6F). Overall, these results demonstrated that TPL@PLGA@F127 exhibited an excellent protective effect in cardiomyocyte survival and on its microstructure.

### TPL@PLGA@F127 enhanced cardiac function after MI

**Fig. 7:**
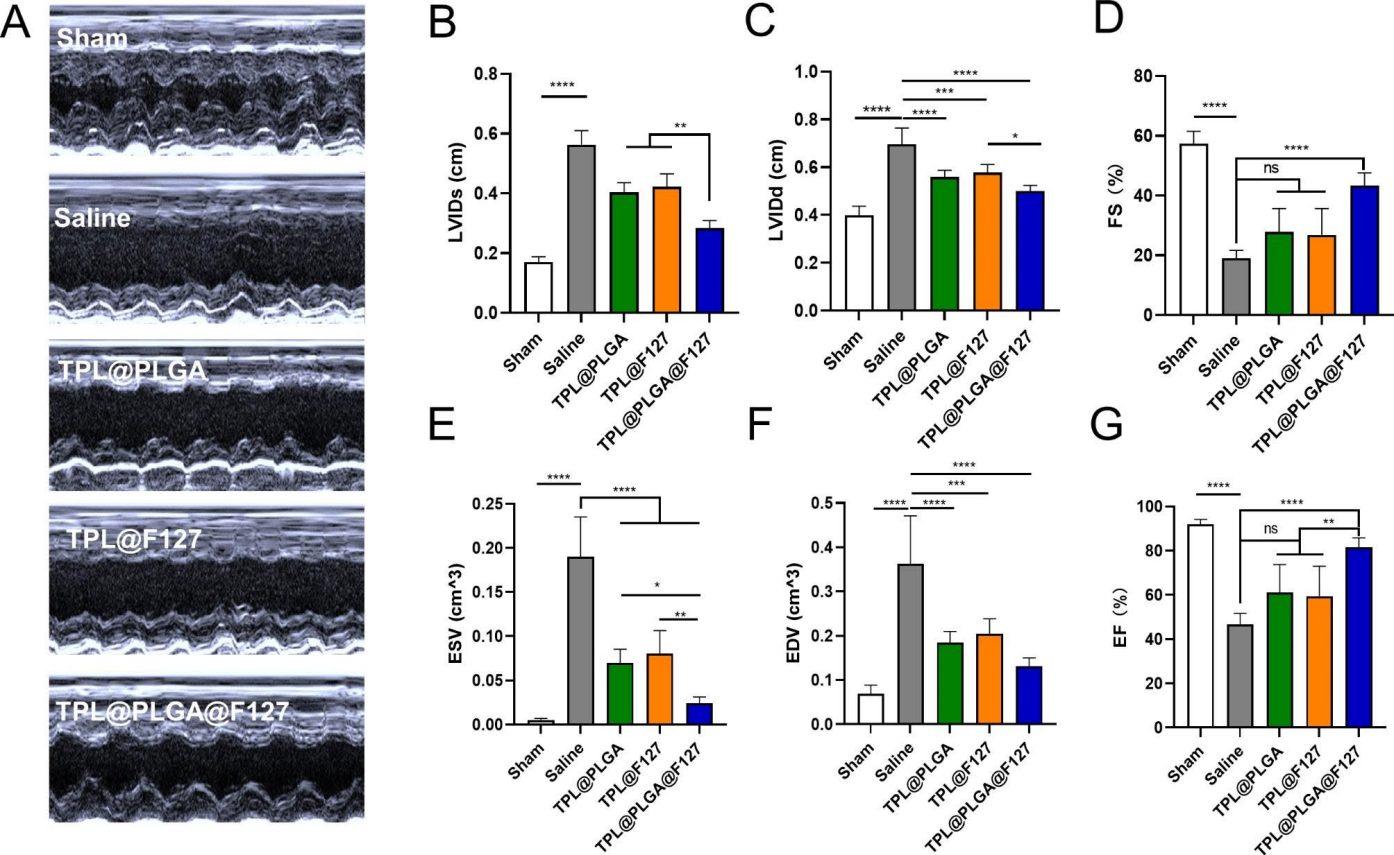
Cardiac function evaluated by echocardiography at days 28 treatment. (**A**) Representative echocardiography images of different groups. (**B**) The left ventricular internal diameter at end-systole (LVIDs). (**C**) The left ventricular internal diameter at end-diastole (LVIDd). (**D**) Fractional shortening (FS). (**E**) End-Systolic Volume (ESV). (**F**) End-diastolic volume (EDV). (**G**) Left ventricle ejection fraction (EF). All bars represent as means ± SD (n = 3). *P < 0.05 and **P < 0.01, ***P < 0.001

As is well established, cardiac function is the most important indicator to assess the therapeutic effect on MI. Therefore, we determined cardiac function by echocardiography at day 28 after the treatment (Fig. 7). Left ventricle ejection fraction (EF, Fig. 7G) and Fractional shortening (FS, Fig. 7D) were increased and the left ventricular internal diameter at end-diastole (LVIDd, Fig. 7C), The left ventricular internal diameter at end-systole (LVIDs, Fig. 7B), End-diastolic volume (EDV, Fig. 7F) and End-Systolic Volume (ESV, Fig. 7E) were decreased by varying degree in the TPL@PLGA, TPL@F127, and TPL@PLGA@F127 groups compared with the saline group (Fig. 7A-G). Among these groups, TPL@PLGA@F127 displayed the best protection of cardiac function, which we believed resulted from the sustainable and stable provision of TPL.

### TPL@PLGA@F127 inhibited myocardial fibrosis

**Fig. 8:**
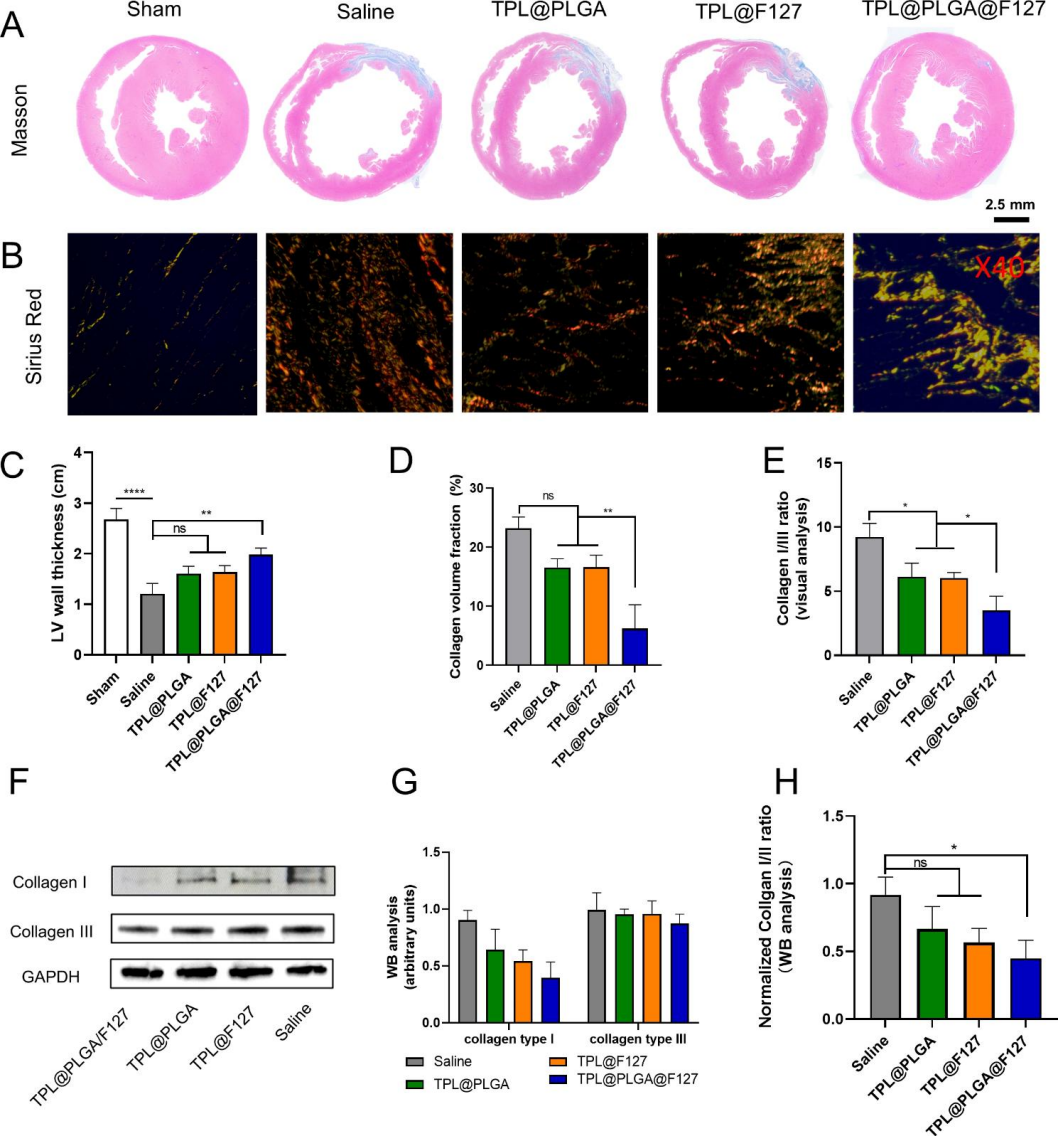
Assessment of myocardial fibrosis by pathological staining and western blot at day 28 post the treatment. (A) Masson trichrome staining at the level of cardiac papillary muscle, the semi-quantitative analysis of LV wall thickness (C) and collagen volume fraction (D), In Masson staining, collagen was stained blue, scale bars of heart cross sections of Masson trichrome staining: 2.5 mm. (B) Sirius Red staining (Sirius red staining was observed at 40 magnification, and scale bars is 0.5 mm. The red staining was collagen, the red and yellow tissues observed were collagen I, while the green tissues observed was collagen III). (E) The semi-quantitative analysis of collagen I/III ratio based on Sirus red staining. (F) the western blot of collagen I and III in myocardial infarction tissue and the semi-quantitative analysis (G&H). All bars represent as means ± SD (n = 3). *P < 0.05 and **P < 0.01, ***P < 0.001

In general, under the stimulation of the inflammatory and immune microenvironment in the infarcted myocardium, myocardial fibrosis appears to be inevitable, contributing to decreased myocardial compliance and extremely poor cardiac function [26]. To identify the therapeutic effect of TPL@PLGA@F127 on myocardial fibrosis, we evaluated the degree of fibrosis by Masson staining and the result showed that the largest volume of collagen and the weakest ventricular thickness were in the saline group. Compared with this, the TPL@PLGA@F127 group displayed a more normal ventricular thickness and less collagen deposition (Fig. 8A, and 8 C-D). The immune process and chronic inflammatory response after MI would generally constantly stimulate the myocardium, resulting in the deposition of various collagens, of which the collagen I and III are the most important and the ratio of collagen I/III is directly proportional to the degree of myocardial fibrosis [27]. Therefore, we performed the Sirus red staining (Fig. 8B) and determined the collagen I/III ratio (Fig. 8E), with the TPL@PLGA@F127 group having a significantly lower collagen I/III ratio than the other groups. To further prove this point, we detected the expression of collagen I and collagen III in infarcted myocardium and the result showed that TPL@PLGA@F127 could significantly decrease the expression of collagen I and the collagen I/III ratio (Fig. 8F-H), which was similar to the result of Sirus red staining. Overall, these results indicated that TPL@PLGA@F127 had the best effects in preventing myocardial fibrosis.

## Discussion

TPL, as a traditional Chinese medicine monomer with numerous biological functions in many diseases, is, however, rarely used in the treatment of cardiovascular diseases, particularly MI, and the evidence of whether TPL can treat MI is insufficient. First, we analyzed the possibility and possible mechanism of TPL against MI by network pharmacology. The results showed that the inflammatory response, negative regulation of apoptotic process, and immune response might be the main mechanisms of TPL against MI (Fig. 1C). Subsequently, we injected TPL intraperitoneally into rats to treat the rats with MI, and found that under this dose condition, half of the rats with successful modelling died within one day after the treatment (Fig. S3). We also observed the toxicity of TPL in normal organs (heart, liver, spleen, lung and kidney) at the day 3 by HE staining (Fig. 4) and found that inflammatory cell infiltration, severe edema, and the destroyed structure in liver and kidney tissue in the TPL (i.p) group, indicating that TPL had the significant hepatotoxicity. We thought that the cause for the death in TPL (i.p.) group mainly were two points, one being the clear hepatotoxicity of TPL, while the other was that the rats were frail after the MI-operation, which led to a low tolerance to TPL. Therefore, intraperitoneal injection might not be the rational TPL administration, and other administration routs should be considered to take full advantage of TPL.

Intramyocardial delivery refers to the direct injection of a drug into the myocardium, which can avoid the systemic toxicity caused by systemic administration. PLGA is a biodegradable copolymer approved by FDA, and PLGA nanoparticles can be used to encapsulate a variety of drugs to increase bioavailability and provide slow and sustained drug release [12]. However, PLGA has the problem of burst release in the early stage, and more importantly, myocardial contraction and diastole make complete PLGA nanoparticle retention in the myocardium difficult by direct intramyocardial injection. F127 is a form of temperature-sensitive hydrogel, and can be converted to gel at body temperature after injected into myocardium, which will promote the intramyocardial retention of F127. Of note, F127 also has an early sustained release effect, such that F127 may be able to avoid the burst release of PLGA nanoparticle. Therefore, based on the temperature-sensitive properties of the F127 gel and slow-release properties of the PLGA nanoparticle and F127 gel, we designed and synthesized the TPL@PLGA@F127 hydrogel platform, which was expected to achieve sustained TPL release and the effects of synergism and detoxification.

Firstly, we identified the characteristics of TPL@PLGA@F127. The results showed that the design of TPL@PLGA@F127 could not only ensure slower and more stably TPL release (Fig. 2D), but also promoted its retention in the myocardium (Fig. 2E), and the design was similar with the strategies promoting stem cell retention in other reports [28]. Subsequently, considering that the result of network pharmacology indicted that TPL might be able to regulate the immune process, depress inflammation and reduce apoptosis, we evaluated the therapeutic effect and mechanism of TPL@PLGA@F127 in following experiments. Previous study indicated that the immunophenotype of macrophages was mainly related to the immune response related to the prognosis of MI [29], thereby, we evaluated the effects of TPL@PLGA@F127 on macrophages. MI rats were treated with saline, TPL@PLGA, TPL@F127, TPL@PLGA@F127, or TPL (i.p.) and the immunofluorescence staining of infarcted myocardium showed that TPL@PLGA@F127 and TPL@F127 had the best effect on immune regulation compared with other groups (Fig. 3C and E). The conventional administration of TPL is by intraperitoneal injection, but this would contribute to the low bioavailability, thus the effect of immunoregulation in the TPL (i.p.) group was not significant although it was better than the saline group. The TPL@PLGA group had a lower drug concentration in the myocardium because of the poor drug retention, while the F127 group did not have the problem of direct drug efflux out of the heart, so the effect of the F127 group was significantly better than the PLGA@F127 group. The TPL@PLGA@F127 group had a lower number of repaired macrophages (M2 subtype) compared with the F127 group, and we believed that this was due to the higher TPL concentration in the heart induced by the extremely rapid release in the F127 group. However, we did not observe a statistically significant difference between these two groups.

The result of network pharmacology indicated the TPL might also be used to treat MI by depressing the inflammatory process. Generally, the chronic inflammatory process post MI has a significant effect on the recovery of infarcted myocardium and it is the basic pathological process of MI, in which many inflammatory cells, including macrophages, will infiltrate in the infarcted myocardium. Macrophages are the main cells which mediate the inflammatory processes, particularly chronic inflammation [30]. CD68 is a surface biomarker expressed by all macrophages [31], on treating MI rats with saline, TPL@PLGA, TPL@F127, or TPL@PLGA@F127, and we observed the effect on the infiltration of the inflammatory cells at day 28 and the expression of inflammatory factors (TNF-α, IL-10) in the infarcted myocardium by immunofluorescence staining (Fig. 5A and B) and western blot (Fig. 5C-F) respectively. The results showed that the TPL@PLGA@F127 group had a more pronounced long-acting inflammatory inhibitory effect compared with the TPL@F127 group. The TPL@F127 group displayed a good immunomodulatory effect by releasing a large amount of TPL early, but the drug release by TPL@F127 could not support a long-acting inflammatory inhibition. By contrast, the TPL@PLGA group had less myocardial residency and the inflammatory inhibition was relatively insignificant. Unlike in the experiments at day 3 assessing the immunomodulatory effects of treatment, we did not include the TPL (ip) group in the inflammatory inhibition assessment at day 28, because from previous experiments the TPL (i.p.) would have significant hepatotoxicity and nephrotoxicity and reduce survival in MI rats, and there was no significant immunomodulatory ability by day 3. These were also the reasons why we did not include TPL (i.p.) in subsequent experiments. Overall, the results of the animal experiments from day 28 demonstrated that TPL@PLGA@F127 exhibited an excellent anti-inflammatory effect for a long period, which was similar with the results of network pharmacology.

The result of network pharmacology indicated the TPL might also be used to treat MI by affecting the apoptosis process. Therefore, we first assessed the survival of cardiomyocyte by performing TUNEL staining and western blot of infarcted myocardium and found that the TPL@PLGA@F127 group had the least TUNEL-positive cell compared with the other groups except the sham group (Fig. 6A and C). Additionally, the results of western blot showed that TPL@PLGA@F127, TPL@PLGA and TPL@PLGA@F127 all could reduce the expression of cleaved caspase 3, with TPL@PLGA@F127 having the greatest effect (Fig. 6D and E). In addition, we evaluated the myocardial microstructure by immunofluorescence staining (Fig. 6B-F). The result showed that TPL@PLGA@F127 could maintain the stable expression of CX43 and α-actinin in myocardial infarction tissue. CX43 is a form of connexin expressed in the intramyocardial tissue and is necessary for normal cardiac conductivity [32]. The anoxic microenvironments in myocardial infarction tissue can contribute CX43 degradation, which may cause arrhythmia [33]. Overall, TPL@PLGA@F127 had an excellent effect in reducing cardiomyocyte apoptosis and in protecting myocardial structure, which also conformed the result of network pharmacology.

Finally, we also assessed the long-term therapeutic effect of TPL@PLGA@F127 by echocardiography at days 28 post MI-operation (Fig. 7) and found that it had the best effect in improving the LVIDs, LVIDd, ESV, and EDV compared with the other groups. To further study the therapeutic effects, we also identified changes of myocardial tissue structure by Masson and Sirius red staining and western blot. In general, following the occurrence of MI, macrophages, neutrophils, and T and B cells are gradually recruited and activated [34], consequently releasing inflammatory factors, reactive oxygen species (ROS), and pro-fibrotic factors, which will activate local resident fibroblasts and induce their differentiation into myofibroblasts. These myofibroblasts induce collagen I/III fibers and excessive extracellular matrix accumulation, which is an underlying pathological characteristic of cardiac fibrosis [27, 35]. In addition, heart chambers will become larger and the ventricular wall thinner because of myocardial fibrosis and infarction, which are the main changes in anatomic structure [36]. In our study, TPL@PLGA@F127 could prevent the dilatation of ventricle and the thinning of ventricular wall, which was the anatomic foundation of the recovery of cardiac function (Fig. 8A and C). In addition, the result of Sirius red collagen staining (Fig. 8B) and western blot (Fig. 8F) showed that TPL@PLGA@F127 has the most significant effect on the decrease of collagen I and the collagen I/III ratio. These results all revealed that TPL@PLGA@F127 has a long-term effect on MI.

## Conclusion

In this study, we developed a form of long-acting hydrogel platform (TPL@PLGA@F127) based on the slow degradation of PLGA, intramyocardial retention of F127 and synergistic effects between them. The results clearly demonstrated that TPL@PLGA@F127 not only displayed promising therapeutic effects on MI, but also attenuated the toxicity of TPL on normal organs. TPL@PLGA@F127 could polarize macrophages toward the anti-inflammatory phenotype (M2 macrophages) within the first 3 days, which could reduce the inflammation process in myocardial infarction more effectively and protect the cardiomyocytes during the early stage. At the same time, TPL@PLGA@F127 could also improve the cardiac function in the long-term. Therefore, we expect that this hydrogel platform will serve as a scientific basis for MI treatment.

## Electronic supplementary material

Below is the link to the electronic supplementary material.

Supplementary Material 1

## Data Availability

All data needed to evaluate the conclusions in the paper are present in the paper and/or the Additional files.

## Abbreviations

BP: Biological process
CC: Cellular components MF:Molecular functions PPI:Protein-protein interaction
CX43: Connexin 43
DCM: Dichloromethane
EE: Encapsulation efficiency
EF: Left ventricle ejection fraction
ESV: End-Systolic Volume
F127: Pluronic F-127 PLGA:Polylactic-co-glycolic acid
FBS: Fetal bovine serum
FDA: The U.S. Food and Drug Administration
FS: Fractional shortening
HPLC: High-performance liquid chromatography
LVIDd: The left ventricular internal diameter at end-diastole
LVIDs: The left ventricular internal diameter at end-systole
MI: Myocardial infarction
PVA: Polyvinyl alcohol
ROS: Reactive oxygen species
SEM: Scanning Electron Microscope
TEM: Transmission Electron Microscope
TPL: Triptolide
