# Supplementary material for: Triptolide with hepatotoxicity and nephrotoxicity used in local delivery treatment of myocardial infarction by thermosensitive hydrogel
Source: J Nanobiotechnology. 2023 Jul 17;21:227. doi: 10.1186/s12951-023-01980-6 (PMC10351172; doi:10.1186/s12951-023-01980-6)
Supplement: Supplementary file 1 — Supplementary Material 1 [file 12951_2023_1980_MOESM1_ESM.docx]

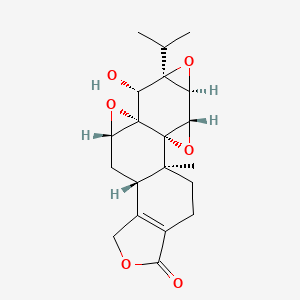


**Fig. S1** The chemical structure of TPL (<https://pubchem.ncbi.nlm.nih.gov/>).

**
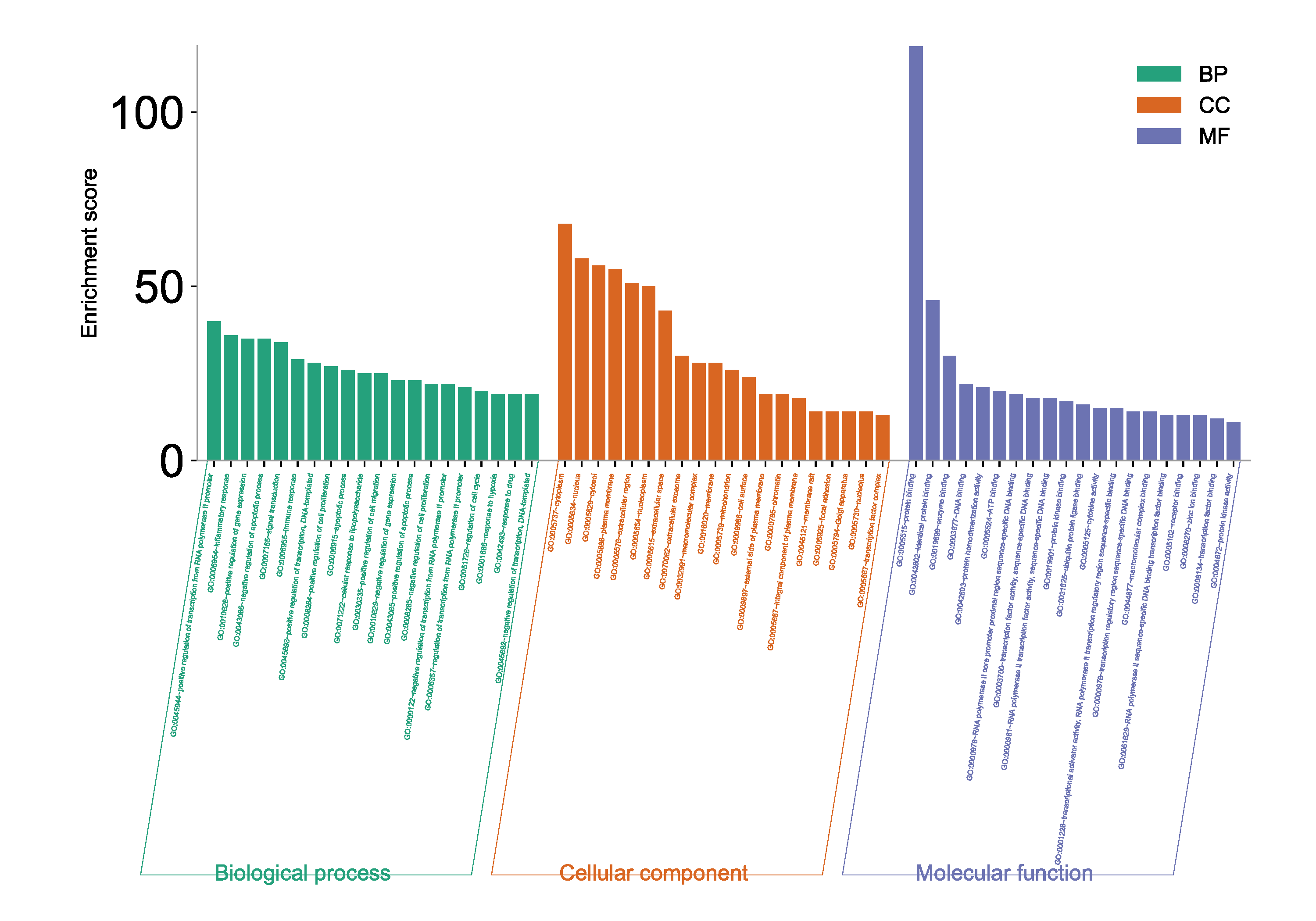
**

**Fig. S2** The GO enrichment analysis of TPL against MI.


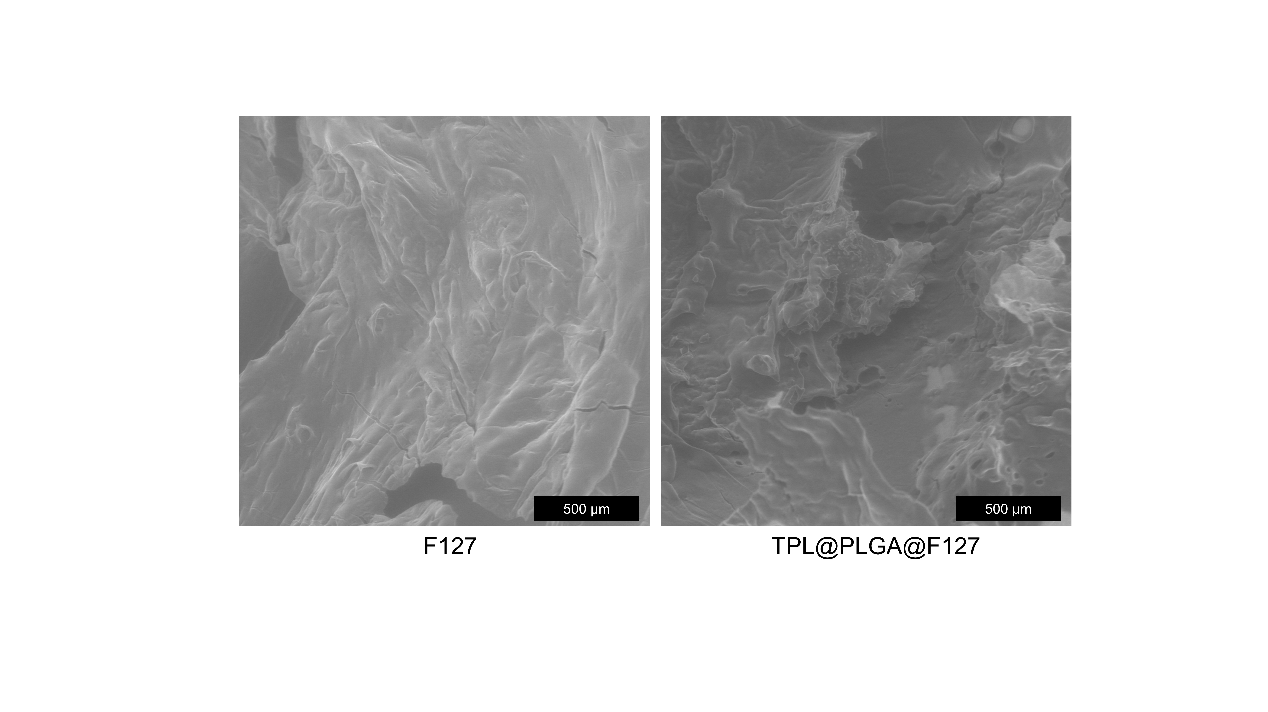


**Fig.S3** The SEM of F127 and TPL@PLGA@F127.

**
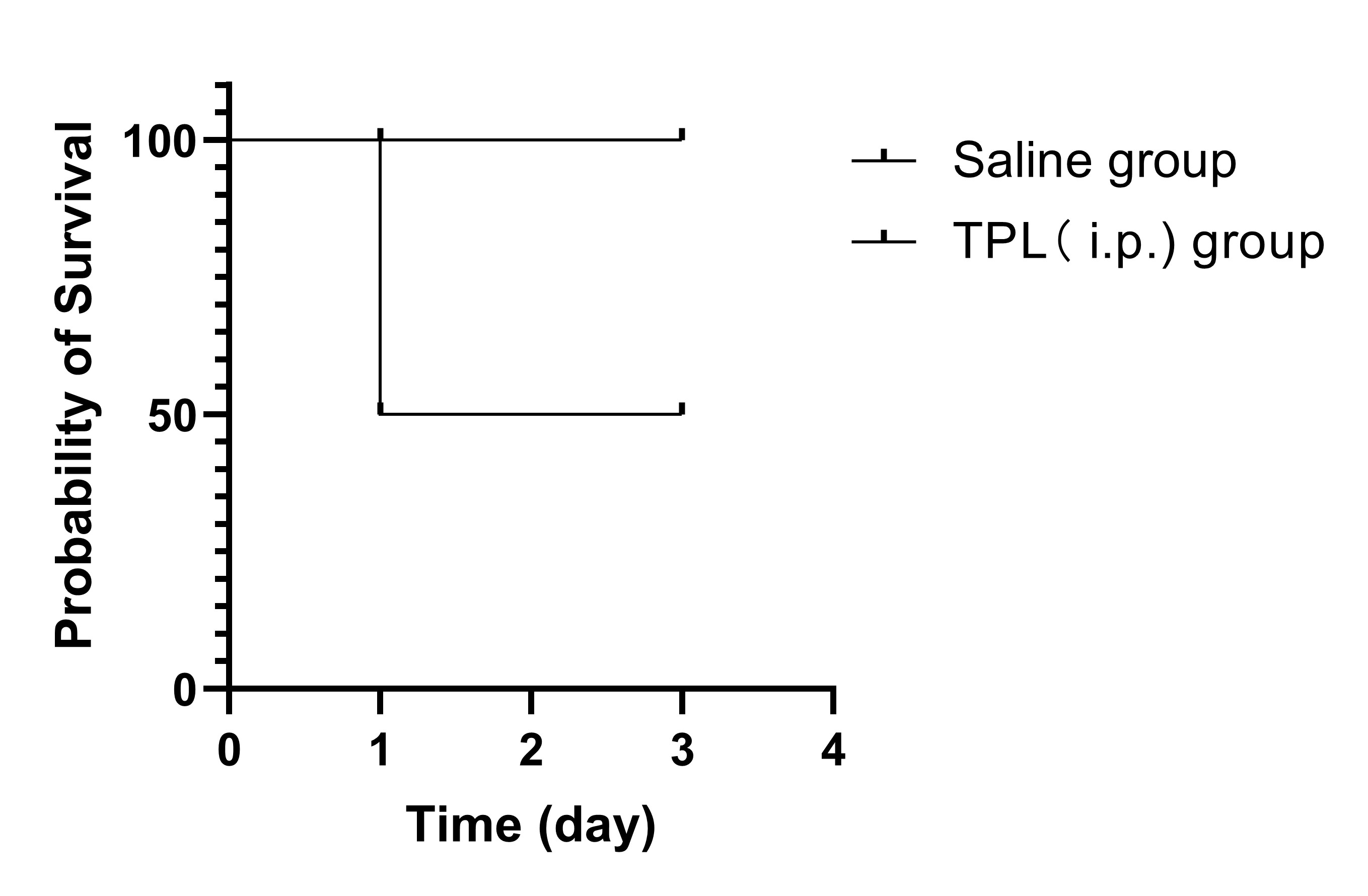
**

**Fig. S4** Survival curves of MI rats after different treatments


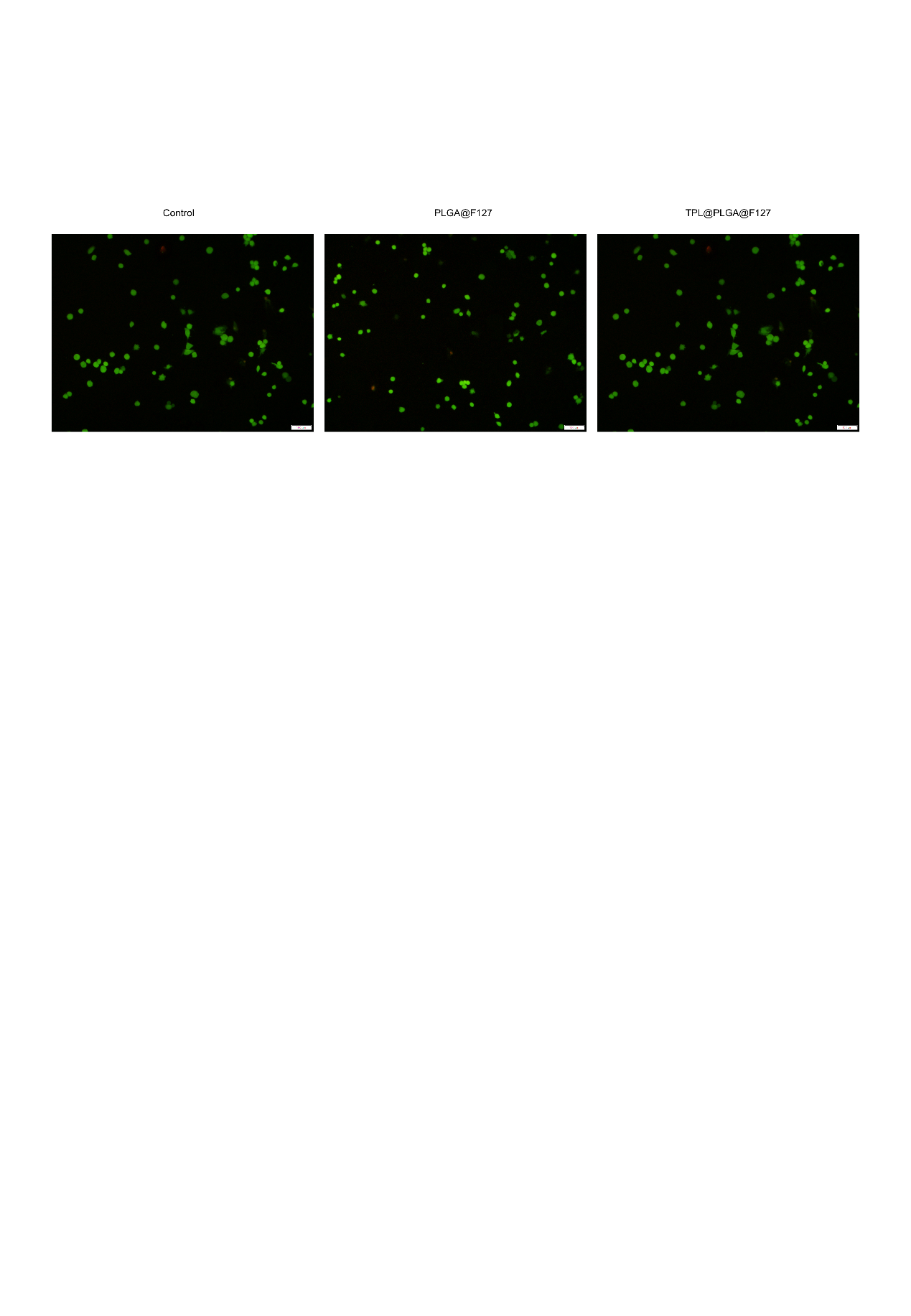


**Fig.S5** The live/dead cells’ staining of H9C2. The green indicates live cells and the red indicates the dead cells.


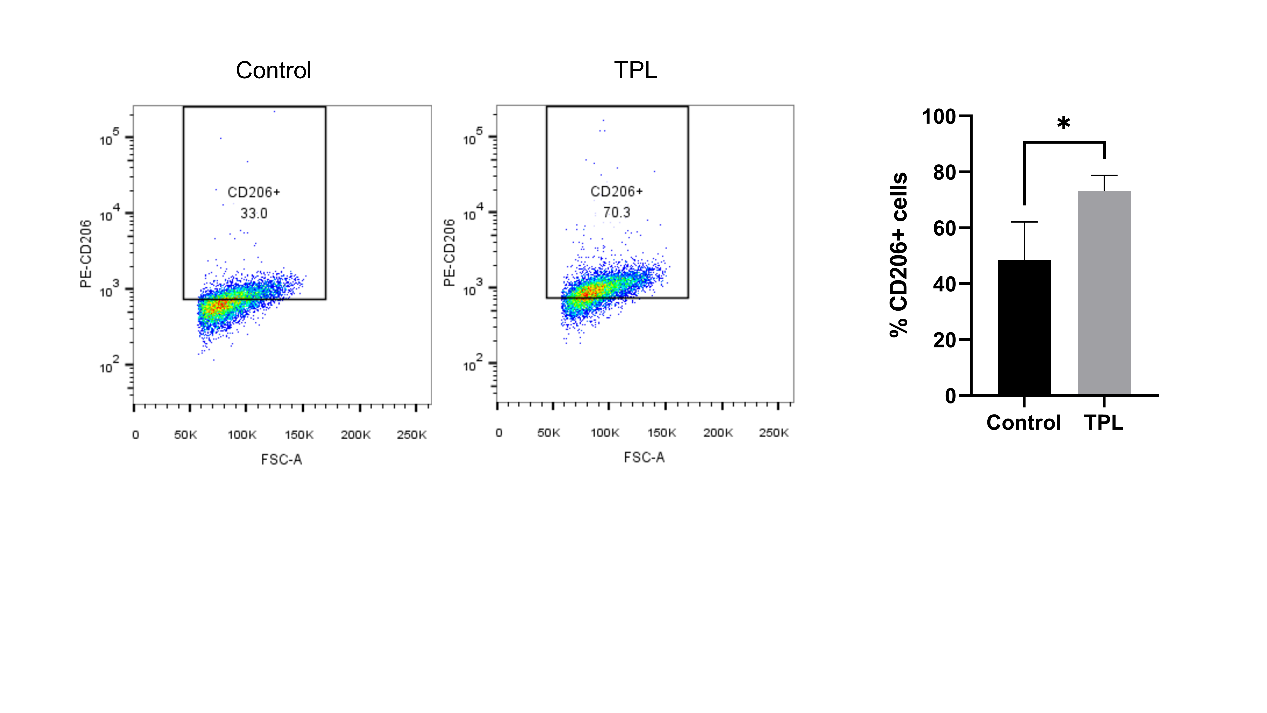


**Fig.S6** the result of flow cytometry about the effect of TPL on macrophages.

**Table S1**. the number of gene related to MI.

| **Database** | **Gene number** |
| --- | --- |
| GeneCards | 1829 |
| Drugbank | 34 |
| OMIM | 208 |
| CTD | 17 |

**Table S2**. the number of gene related to TPL.

| **Database** | **Gene number** |
| --- | --- |
| TCMSP | 32 |
| GeneCards | 219 |

**Table S3** the 126 interaction genes between TPL and MI related genes

| BAX | CD14 | IL2 | SP1 | PLAU |
| --- | --- | --- | --- | --- |
| BCR | TGFB1 | NOS2 | NR3C1 | IL37 |
| CEBPA | PTEN | THBS1 | MAPK3 | CXCR2 |
| ESR1 | IL4 | KDR | BSG | CCR3 |
| IRF1 | MMP3 | CCL3 | EZH2 | XRCC1 |
| JAK2 | BCL2 | PIK3CG | HSPA4 | TNFSF11 |
| KIT | PTGS2 | STAT1 | MAPK8 | CYP2E1 |
| STAT3 | CASP3 | GDF15 | TNFSF10 | HSPA5 |
| TERT | C3 | MAPK14 | SOD2 | CCR7 |
| PLAUR | MMP2 | CDKN1A | BIRC5 | MMP7 |
| TP53 | HIF1A | PARP1 | CD80 | CXCL2 |
| IL6 | CCR5 | MDM2 | SOCS1 | CNR2 |
| TNF | MYC | TEK | MCL1 | XRCC5 |
| APOE | CXCR4 | MAPK1 | NFKB1 | SHH |
| MMP9 | CCL11 | CXCL10 | IL2RB | CCL17 |
| MIR155 | CCL5 | JUN | CAV1 | PRKDC |
| IL1B | ALOX5 | GAPDH | TH | RELA |
| VEGFA | ANXA5 | HSPA8 | HSPA1A | MMP13 |
| CXCL8 | MIR204 | CASP8 | CD274 | REST |
| IFNG | TLR2 | IL5 | TNFRSF10B | ARNT |
| IDH1 | MIR21 | XIAP | CFLAR | XRCC6 |
| CCL2 | CSF2 | CASP9 | CDKN3 | MET |
| ICAM1 | AKT1 | LAMP2 | CD86 | ADAMTS4 |
| CSF3 | CD40 | FOS | APC | XK |
| CXCL12 | PTGS1 | NFE2L2 | GSK3B | SOX9 |
| ADAM10 |  |  |  |  |

**Table S4**. the TOP 20 biological processes of GO enrichment analysis.

| **GO term** | **Subgroup** | **Enrichment score** |
| --- | --- | --- |
| GO:0045944~positive regulation of transcription from RNA polymerase II promoter | Biological process | 40 |
| GO:0006954~inflammatory response | Biological process | 36 |
| GO:0010628~positive regulation of gene expression | Biological process | 35 |
| GO:0043066~negative regulation of apoptotic process | Biological process | 35 |
| GO:0007165~signal transduction | Biological process | 34 |
| GO:0006955~immune response | Biological process | 29 |
| GO:0045893~positive regulation of transcription, DNA-templated | Biological process | 28 |
| GO:0008284~positive regulation of cell proliferation | Biological process | 27 |
| GO:0006915~apoptotic process | Biological process | 26 |
| GO:0071222~cellular response to lipopolysaccharide | Biological process | 25 |
| GO:0030335~positive regulation of cell migration | Biological process | 25 |
| GO:0010629~negative regulation of gene expression | Biological process | 23 |
| GO:0043065~positive regulation of apoptotic process | Biological process | 23 |
| GO:0008285~negative regulation of cell proliferation | Biological process | 22 |
| GO:0000122~negative regulation of transcription from RNA polymerase II promoter | Biological process | 22 |
| GO:0006357~regulation of transcription from RNA polymerase II promoter | Biological process | 21 |
| GO:0051726~regulation of cell cycle | Biological process | 20 |
| GO:0001666~response to hypoxia | Biological process | 19 |
| GO:0042493~response to drug | Biological process | 19 |
| GO:0045892~negative regulation of transcription, DNA-templated | Biological process | 19 |
